# Mediastinal Yolk Sac Tumor With Solitary Adenocarcinoma in Subcarinal Nodal Metastases

**DOI:** 10.1016/j.atssr.2023.09.005

**Published:** 2023-09-13

**Authors:** Ryosuke Tokuda, Masanori Shimomura, Satoru Okada, Shunta Ishihara, Aya Miyagawa-Hayashino, Hiroshi Watanabe, Noriyuki Tanaka, Masayoshi Inoue

**Affiliations:** 1 Division of Thoracic Surgery, Department of Surgery, Graduate School of Medical Science, Kyoto Prefectural University of Medicine, Kyoto, Japan; 2 Department of Surgical Pathology, Graduate School of Medical Science, Kyoto Prefectural University of Medicine, Kyoto, Japan

## Abstract

Germ cell tumors with somatic-type solid malignancy are mostly attributed to the malignant transformation of teratomas. Herein, we describe the case of a 41-year-old man with mediastinal yolk sac tumor who presented with adenocarcinoma in nodal metastases. Computed tomography revealed enlarged subcarinal lymph nodes and an anterior mediastinal tumor, which was diagnosed as a yolk sac tumor by percutaneous biopsy. The remaining tumor was resected after chemotherapy. No viable cells were observed in the primary tumor; however, adenocarcinoma was detected in the subcarinal lymph nodes. Mediastinal yolk sac tumors can be associated with metastatic adenocarcinoma in locoregional lymph nodes.

A germ cell tumor with somatic-type solid malignancy (GCT-STM) is defined as a germ cell tumor with a component of a malignant neoplasm resembling that observed at somatic sites, with most of the somatic components arising from the malignant transformation of a teratoma in the primary tumor.^1^ We encountered an unusual case of a yolk sac tumor of mediastinal origin without a teratoma component in the primary tumor but manifested with adenocarcinoma in the mediastinal metastatic lesion.

A 41-year-old man was referred to our hospital with an anterior mediastinal mass detected on chest computed tomography (CT), which revealed an anterior mediastinal tumor, 15 cm in diameter, and enlarged subcarinal lymph nodes, 3.2 cm in short-axis diameter (Figure 1A). Positron emission tomography/CT revealed high fluorodeoxyglucose uptake (Figure 1B). The serum α-fetoprotein (AFP) and human chorionic gonadotropin β levels were elevated (253 ng/mL and 16 ng/mL, respectively). CT-guided needle biopsy revealed a Schiller-Duval body with a perivascular arrangement (Figure 2A) and positive immunohistologic staining for Sal-like protein 4 (SALL4), a specific marker for germ cell tumors (Figure 2B), leading to a diagnosis of yolk sac tumor. After 5 courses of chemotherapy (etoposide, ifosfamide, cisplatin), the serum AFP and human chorionic gonadotropin β levels normalized. The tumor and subcarinal lymph nodes reduced to 6.7 cm and 1.5 cm, respectively (Figures 1C, 1D). Although the treatment response was partial, the main tumor and subcarinal nodes were still detected as lesions on CT.

**Figure 1:**
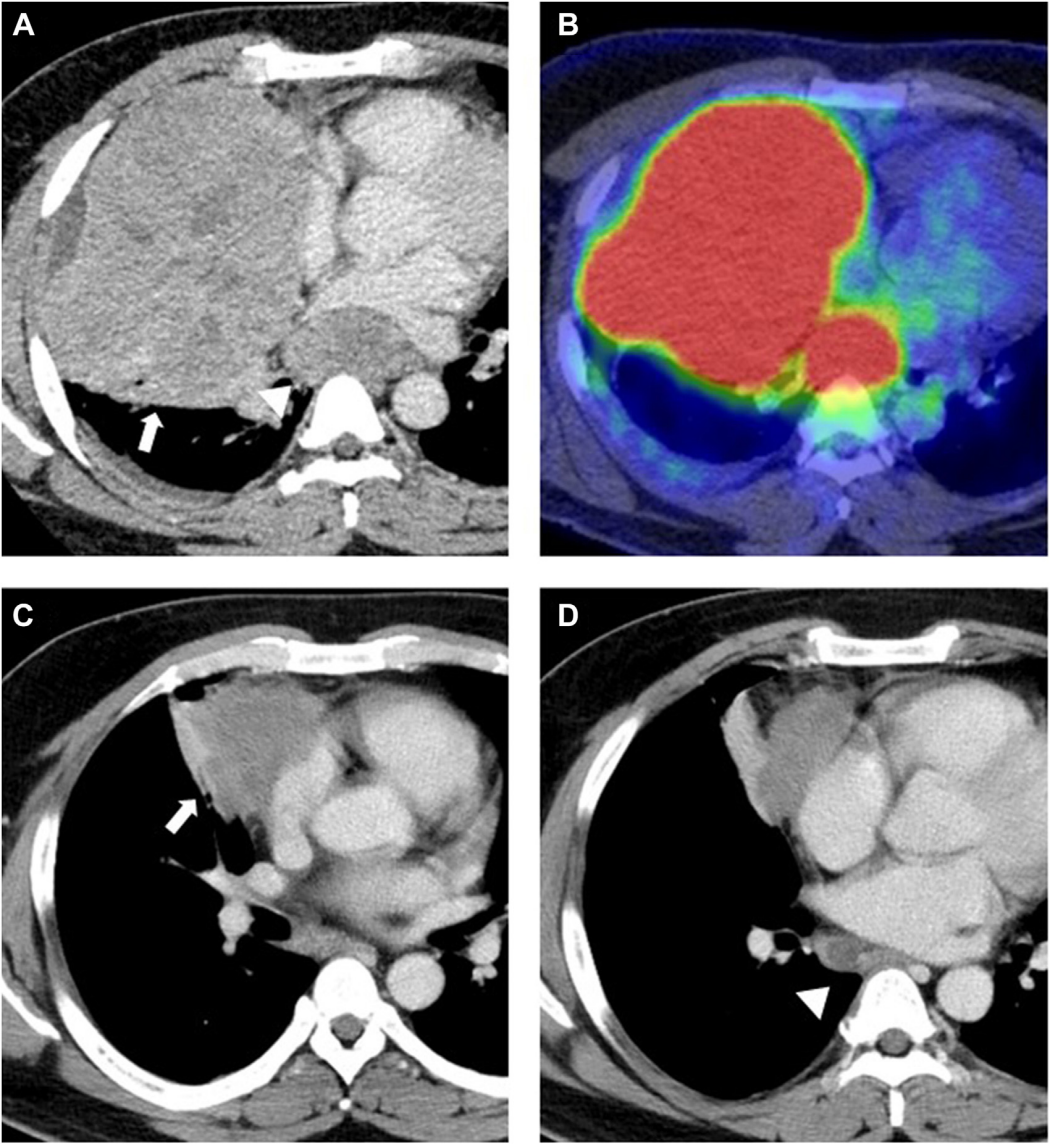
Preoperative radiographic findings. (A) Contrast-enhanced computed tomography (CT) revealing a mediastinal tumor protruding into the right thoracic cavity (arrow) and enlarged subcarinal lymph nodes (arrowhead). (B) Positron emission tomography/CT revealing a high fluorodeoxyglucose uptake (28.7 and 22.4 in the main tumor and subcarinal lymph nodes, respectively). (C, D) CT revealing shrinking mediastinal tumor (arrow) and subcarinal lymph nodes (arrowhead) after chemotherapy.

**Figure 2:**
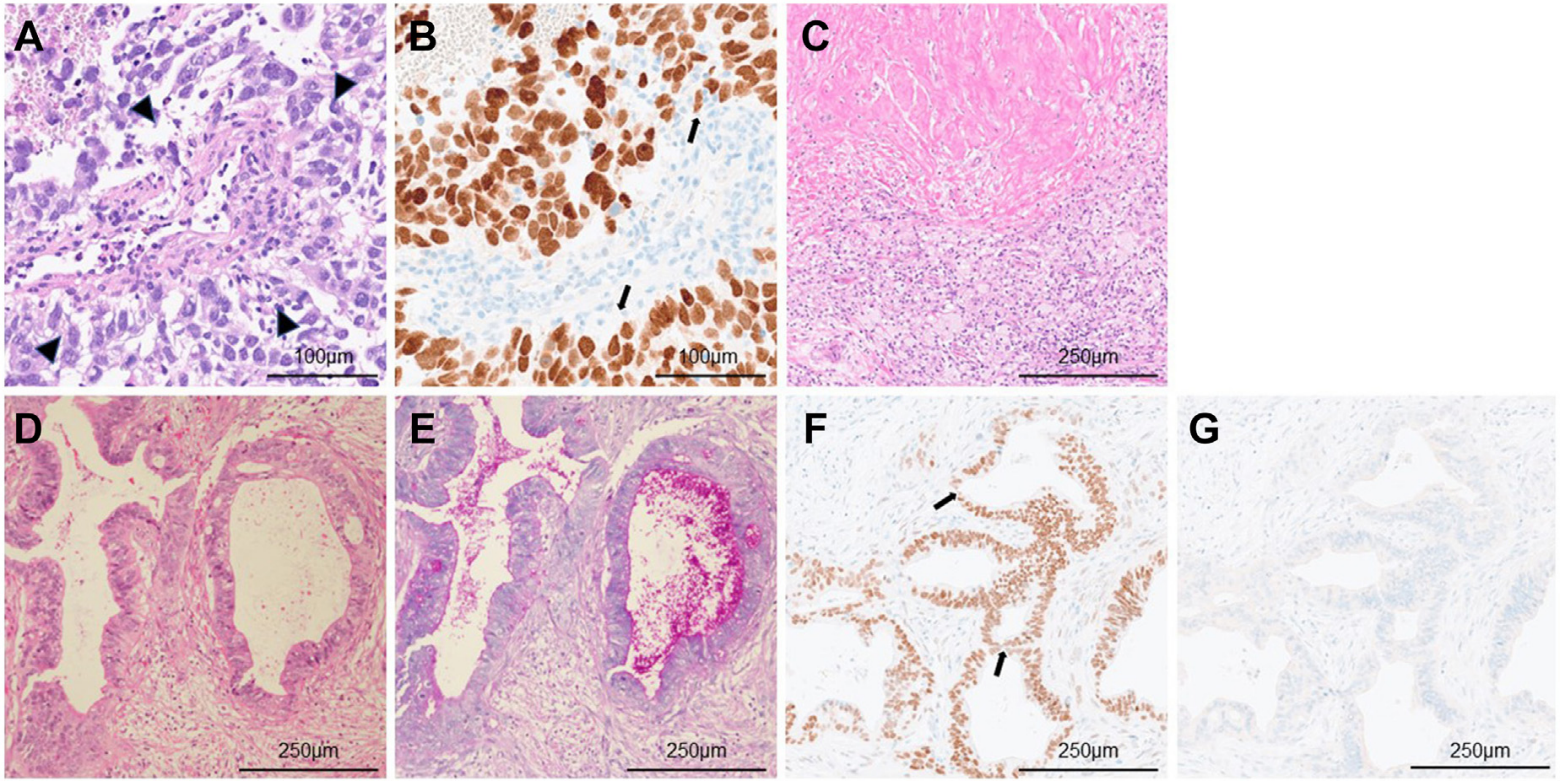
Microscopic findings. (A) Tumor cells in the needle biopsy specimen reveal a Schiller-Duval body with a perivascular arrangement (arrowheads; hematoxylin and eosin [H&E]), and (B) tumor cells are immunohistochemically positive for Sal-like protein 4 (arrows). (C) Coagulation necrosis, fibrosis, and accumulation of foamy macrophages are observed in the resected mediastinal tumor. No viable malignant cells are found (H&E). (D) Tumor cells of the resected subcarinal lymph nodes are carcinoma with atypical cells showing tubular proliferation (H&E), and (E) periodic acid–Schiff staining confirms the presence of intracytoplasmic mucin. On immunohistochemical staining, tumor cells are (F) positive for Sal-like protein 4 (arrows) and (G) negative for thyroid transcription factor 1. (A, B, original magnification ×200; C-G, original magnification ×100.)

Surgical resection of the residual tumor was performed by a median sternotomy with anterolateral thoracotomy through the right third intercostal space. An extended resection of the tumor was performed as it invaded the right middle lobe, pericardium, and phrenic nerve. The pericardium was reconstructed, and diaphragmatic plication was performed. Subcarinal lymph node dissection was attempted from the thoracic cavity after the middle lobe resection. Assistance by the surgeon’s manual retraction under the subcarinal nodes (called the hand-assisted anterolateral approach) enabled the dissection of the subcarinal lymph nodes (Video). The postoperative course was uneventful, and the patient was discharged on day 13.

Histopathologic findings revealed no viable malignant cells (Figure 2C). In contrast, the carcinoma was detected in the subcarinal lymph nodes (Figure 2D). Periodic acid–Schiff staining confirmed the presence of intracytoplasmic mucin (Figure 2E), suggesting that carcinoma cells in the subcarinal lymph nodes were of adenocarcinoma rather than germ cell tumors, such as glandular yolk sac tumors or embryonal carcinomas. On immunohistochemistry, tumor cells were positive for SALL4 as in the mediastinal yolk sac tumor at biopsy, and thyroid transcription factor 1 was negative (Figures 2F, 2G). Thus, the adenocarcinoma of the subcarinal lymph nodes was presumed to have originated from a yolk sac tumor in the mediastinum. Adjuvant chemotherapy was administered postoperatively because of the presence of viable cells in the subcarinal lymph nodes. No recurrence was observed 13 months after surgery.

## Comment

GCT-STM is rare, accounting for approximately 2% of all germ cell tumors,^2^ but it is relatively more common in mediastinal tumors, accounting for 11% to 29% of mediastinal germ cell tumors in adults.^3^ Although GCT-STM is resistant to chemotherapy and has a poor prognosis, complete resection of the tumor can improve prognosis.^4^

Germ cell tumors occasionally metastasize to the subcarinal lymph nodes. Thus, an appropriate approach should be considered for complete resection of both anterior mediastinal tumors and enlarged subcarinal lymph nodes. The general approach to subcarinal lymph nodes through a median sternotomy is a transpericardial approach.^5^ We used the surgical view of anterolateral thoracotomy, which was necessary for lobectomy, to resect the subcarinal lymph nodes from the thoracic cavity. By manually retracting the enlarged lymph nodes from the dorsal to the ventral side, it was possible to dissect them from the bronchus and inferior pulmonary vein. We were able to obtain a sufficient surgical view because the enlarged lymph nodes were located peripherally around the right main bronchus after middle lobectomy.

In this case, the primary tumor was diagnosed as yolk sac tumor preoperatively. However, the primary lesion may have been a mixed mediastinal germ cell tumor, including a yolk sac tumor, because the volume of the biopsy sample was limited. The following 3 hypotheses can be raised about the cause of adenocarcinoma in the subcarinal lymph nodes: (1) the primary tumor was a mediastinal mixed germ cell tumor of the yolk sac with teratoma, and the teratoma component became malignant and manifested adenocarcinoma in the metastases; (2) the adenocarcinoma component of GCT-STM caused by the mediastinal yolk sac tumor metastasized to the subcarinal lymph nodes; and (3) the mediastinal yolk sac tumor metastasized to the subcarinal lymph nodes, where it became malignant. As the primary tumor showed a pathologic complete response to chemotherapy, it is unlikely that teratomas or GCT-STMs resistant to chemotherapy were present. Both the yolk sac tumor in the primary mediastinal lesion and adenocarcinoma in the subcarinal lymph nodes resected were SALL4 positive and AFP negative. These findings suggest that the yolk sac tumor had transformed into an adenocarcinoma at the metastatic site. Among primary germ cell tumors of the testis, adenocarcinoma originating from the yolk sac tumor reportedly metastasized after chemotherapy.^6^ This yolk sac tumor of mediastinal origin manifested as a solitary adenocarcinoma in a metastatic lesion. Thus, mediastinal yolk sac tumors may be associated with metastatic adenocarcinoma in the locoregional lymph nodes during treatment.
