# Supplementary material for: Mediastinal Yolk Sac Tumor With Solitary Adenocarcinoma in Subcarinal Nodal Metastases
Source: Ann Thorac Surg Short Rep. 2023 Sep 13;2(1):108–11. doi: 10.1016/j.atssr.2023.09.005 (PMC11708738; doi:10.1016/j.atssr.2023.09.005)
Supplement: Legend for Video [file mmc1.docx]

**Supplementary Video**: Surgical resection of the residual mediastinal tumor and subcarinal lymph nodes via median sternotomy with anterolateral thoracotomy via the right third intercostal space.
